# The expression and clinical significance of UHRF1 in soft tissue sarcomas and its prognostic value

**DOI:** 10.1097/MD.0000000000038393

**Published:** 2024-06-07

**Authors:** Qiang Shu, XiaoLing Liu, Xing Xiang, Xu Bo

**Affiliations:** a Department of Hepatobiliary Surgery, Neijiang First People’s Hospital affiliated to Chongqing Medical University, Neijiang, China; b Department of Infection Management, Neijiang Hospital of Traditional Chinese Medicine affiliated to Chengdu University of Traditional Chinese Medicine, Neijiang, China.

**Keywords:** computational biology, kinesin, prognosis, soft tissue sarcoma

## Abstract

To explore the expression and prognostic value of UHRF1 gene in soft tissue sarcoma (STS) and its related molecular mechanism. The expression data and clinicopathological parameters of STS were downloaded from the Cancer Genome Atlas (TCGA). The expression level of UHRF1 in STS and adjacent tissues and its relationship with clinicopathological characteristics were analyzed. The expression level of UHRF1 in STS tissues was significantly higher than that in paracancerous tissues (*P* < .001), and the overall survival (OS) time of patients with high UHRF1 expression was significantly shorter than that of patients with low UHRF1 expression (*P* = .002). The expression of UHRF1 was correlated with tumor necrosis, histological type and metastasis, and the differences were statistically significant (*P* = .013; *P* = .001; *P* = .002). The area ratio under receiver operating characteristic (ROC) curve between STS tissue and adjacent tissue of UHRF1 expression was 0.994. Number of tumors (HR = 0.416, 95%CI = 0.260–0.666, *P* < .001), depth of tumor (HR = 2.888, 95%CI = 0.910–9.168, *P* = .033), metastasis (HR = 2.888, 95% CI = 1.762–4.732, *P* < .001), residual tumor (HR = 2.637, 95% CI = 1.721–4.038, *P* < .001) and UHRF1 expression (HR = 1.342, 95% CI = 1.105–1.630, *P* = .003) were significantly associated with OS, and high expression of UHRF1 (HR = 1.387, 95%CI = 1.008–1.907, *P* = .044) was an independent risk factor for the prognosis of STS patients. The results of the nomogram exhibited that UHRF1 expression level had a significant effect on the total score value. GSEA enrichment analysis suggested that UHRF1 was involved in 14 signaling pathways regulating mRNA spliceosome, cell cycle, P53 signaling pathway were identified. Single sample gene set enrichment analysis (ssGSEA) exhibited that the expression of UHRF1 in STS was positively correlated with the level of Th2 cell infiltration, and negatively correlated with plasmacytoid dendritic cells (pDC), natural killer cells (NK), Eosinophils, Mast cells, etc. UHRF1 expression is involved in the immune microenvironment of HCC and affects the occurrence and development of HCC. UHRF1 is highly expressed in STS tissues. It is involved in the regulation of multiple tumor-related signaling pathways and immune cell microenvironment, suggesting that UHRF1 may be a potential molecular marker for prognosis prediction and targeted therapy of STS patients.

## 1. Introduction

Sarcomas originate from mesenchymal tumors, which account for 1% of all solid tumors in adults and 15% of all solid tumors in Children and youth.^[1]^ Although sarcomas are divided into various pathological subtypes, they are currently mainly classified as soft tissue sarcoma (STS) and primary osteosarcoma (PBS).^[2]^ STS is a rare malignancy that makes up about 1% of all malignancies.^[3]^ According to a report in Cancer Statistics 2022, approximately 13,190 new cases of STS were diagnosed in the United States, resulting in approximately 5130 deaths.^[4]^ The most widely pathological types of STS are liposarcoma, leiomyosarcoma, undifferentiated pleomorphic sarcoma (UPS), myxosarcoma, Malignant Peripheral Nerve Sheath Tumors (MPNST), and synovial sarcoma, accounting for about 65% of all STS.^[5]^ STS usually occurs in the extremities or retroperitoneum, and the chief complaint of most patients is a mass with pain.^[6]^10% of STS are accompanied by metastases at the time of diagnosis, the most common site of metastases being the lungs. When patients with STS have distant metastases, the 5-year survival rate drops to 15%.^[7]^ Therefore, these important measures such as early diagnosis, treatment and prognostic monitoring have been used to improve the survival of STS patients on account of its rarity and diversity, the early diagnosis of STS is still very difficult except by histopathology and immunohistochemistry. With the deepening of cancer research, people gradually comprehend the exact mechanism of cancer occurrence, and identify more and more molecular markers that predict prognosis, which can frequently be applied to tumor-targeted therapy, so as to design corresponding targeted therapeutic drugs.^[8]^ Further exploration of the pathogenesis of STS and search for highly sensitive molecular markers is of great significance for the early diagnosis, timely treatment and prognosis prediction of STS.

UHRF1, known as NP95 or ICBP90, is a multifunctional protein with ubiquitin-like plant homeodomains and ring finger domains that play important roles in DNA methylation and histone methylation, DNA damage repair, and cell proliferation, cycle, and apoptosis.^[9]^ Research has shown that UHRF1 is an oncogene, and UHRF1 protein levels are abnormally elevated in varieties of malignant tumor tissues and cells, promoting cancer cell progression. Furthermore, UHRF1 is being considered as a potential biomarker and therapeutic target in cancer therapy.^[10,11]^ For instance, large-scale cancer genomic data analysis showed that patients with lung adenocarcinoma or acute myeloid leukemia with high levels of UHRF1 expression had significantly shorter survival times.^[12,13]^ Overexpression of UHRF1 can accelerate the proliferation and migration of prostate cancer cells and inhibit the apoptosis of cancer cells.^[14]^ UHRF1 regulates the growth of breast cancer cells through estrogen signaling, and low expression of UHRF1 in vivo and in vitro can significantly inhibit the growth of breast cancer cells.^[15]^ Moreover, UHRF1 overexpression accelerates the metastasis of colorectal cancer.^[16]^ As yet, there are relatively few research reports about UHRF1 in STS at home and abroad. Hence, the expression level and prognostic value of UHRF1 gene in STS were analyzed by bioinformatics method, and its potential molecular mechanism was investigated in this study.

## 2. Materials and methods

### 2.1. Data acquisition

Download STS mRNA expression data from the official website of the Cancer Genome Atlas (TCGA) database (https://www.cancer.gov/ccg/research/genome-sequencing/tcga), which included 263 STS tissues and 2 paracancer tissues. The relevant clinicopathological data (including age, gender, Tumor multifocal, tumor necrosis, residual tumor, tumor depth, histological type, metastasis, radiotherapy and UHRF1 expression level) were downloaded. The correlation between UHRF1 expression and clinicopathological factors was analyzed. From Gene Expression Omnibus (GEO) database (https://www.ncbi.nlm.nih.gov/geo/) to download GSE2719 data sets, platform for GPL96, type of expression profile chip, including 39 cases of STS and 15 cases of adjacent tissue, validation UHRF1 in STS and its distribution in the tissue adjacent to carcinoma.

### 2.2. Validation of UHRF1 expression in STS and paracancerous tissues

The “Limma” toolkit in R software (4.0.4) was used to analyze the relationship between UHRF1 expression levels in STS and adjacent tissues, as well as the correlation between UHRF1 and co-expression of immune-related genes.

### 2.3. Kaplan–Meier method analyze the effect of UHRF1 expression level on the survival status of STS patients

According to the median UHRF1 expression level, STS patients were divided into high and low UHRF1 expression groups. Subgroup analysis of STS patients was performed on the basis of tissue type. Kaplan–Meier method was used to analyze the survival status of patients with high UHRF1 expression and low UHRF1 expression.

### 2.4. Construct nomogram

The relevant clinicopathological factors downloaded from the TCGA database were incorporated into the prediction model using the toolkits in R software (4.0.4) such as “Hmisc,” “lattice,” “Formula,” “ggplot2,” “foreign,” and “rms” to construct the nomogram and calibration curve for predicting the prognosis of STS patients.

### 2.5. Gene set enrichment analysis (GSEA)

We used standardized RNA-Seq data obtained from TCGA to enrich gene sets to analyze the molecular signaling pathways potentially regulated by UHRF1 in the development of STS. On the basis of the 75% threshold, GSEA software (v4.1.0) was adopted to analyze the metabolic pathways and biological processes between high and low expression of UHRF1. The GESA parameter setting selected c2KEGG gene set (c2.cp.kegg, V7.5.symbols.gmt), and the number of gene set permutations was set to 1000. The enrichment results with standardized *P* < .05 and error discovery rate < 0.25 were statistically significant.

### 2.6. Constructed volcano map

The clusterPorfiler R package was utilized to analyze significantly differentially expressed genes (DEG) between STS and paracancer tissue, the ggplot2 package was utilized to visualize the volcanic map of the difference analysis results. The adjusted threshold was calculated by Benjamini-Hochberg method (*P* ≤ .05 and |logFC|≥1.5).

### 2.7. Construction and analysis of protein-protein interaction networks

Refer to STRINGV11 database (https://cn.string-db.org)^[17]^ to analyze the protein-protein interaction (PPI) network of UHRF1 expression. The PPI network was visualized by Cytoscape_v3.10.0 software. MCODE toolkit was used to screen out Hub gene in PPI network.^[18]^

### 2.8. Single sample gene set enrichment analysis (ssGSEA)

Through literature review, 24 marker genes of related immune cells were identified.^[19]^ The infiltration of these immune cells in STS was analyzed by ssGSEA. Spearman correlation method was applied to analyze the correlation degree between UHRF1 expression and immune cells. Wilcoxon rank sum test was applied to compare the infiltration of immune cells in UHRF1 samples with high and low expression.

### 2.9. Statistics process

SPSS software (version 25.0) was utilized to analyze the data. The enumeration data were expressed by the number of cases (percentage) [n (%)], and the difference between groups was compared by χ^2^ test. The overall survival (OS) rate was analyzed by Kaplan–Meier method, and the risk factors affecting the prognosis of patients were analyzed by Cox proportional hazards model. Graphs were analyzed with GraphPad Prism Version 9.02. *P* < .05 was considered as statistically significant.

## 3. Results

### 3.1. Relationship between UHRF1 expression and clinicopathological characteristics

The clinicopathological Characteristics of STS patients were downloaded from the TCGA database (Table 1) to analyze the relationship with UHRF1 expression level. The results revealed that the expression level of UHRF1 was significantly related to tumor necrosis (*P* = .013), histological type (*P* < .001), and metastasis (*P* = .046).

**Table 1:** Relationship between UHRF1 expression level and clinicopathological characteristics of soft tissue sarcoma patients.

Characteristics	Low expression of UHRF1	High expression of UHRF1	*P* value
n	131	132	
Age, n (%)			.295
<=≤60	69 (26.2%)	61 (23.2%)	
> 60	62 (23.6%)	71 (27%)	
Gender, n (%)			.669
Female	70 (26.6%)	74 (28.1%)	
Male	61 (23.2%)	58 (22.1%)	
Tumor multifocal, n (%)			.544
Yes	18 (7.5%)	22 (9.2%)	
No	100 (41.8%)	99 (41.4%)	
Tumor necrosis, n (%)			.013
Moderate necrosis & Extensive necrosis	25 (13.7%)	49 (26.8%)	
Focal necrosis & No necrosis	57 (31.1%)	52 (28.4%)	
Residual tumor, n (%)			.962
R0	80 (34%)	77 (32.8%)	
R1&R2	40 (17%)	38 (16.2%)	
Tumor depth, n (%)			.946
Deep	91 (43.5%)	97 (46.4%)	
Superficial	10 (4.8%)	11 (5.3%)	
Histological type, n (%)			.001
Leiomyosarcoma	54 (20.7%)	52 (19.9%)	
Dedifferentiated liposarcoma	34 (13%)	24 (9.2%)	
Pleomorphic sarcoma	14 (5.4%)	38 (14.6%)	
Myxofibro sarcoma	12 (4.6%)	13 (5%)	
Synovial sarcoma	9 (3.4%)	1 (0.4%)	
Malignant peripheral nerve sheath tumors	6 (2.3%)	4 (1.5%)	
Desmoid tumor	0 (0%)	2 (3.3%)	
Metastasis, n (%)			.002
Yes	15 (8.4%)	44 (24.6%)	
No	60 (33.5%)	60 (33.5%)	
Radiation therapy, n (%)			.090
Yes	32 (12.5%)	46 (17.9%)	
No	94 (36.6%)	85 (33.1%)	

### 3.2. UHRF1 showed high expression level in STS tissue

**Figure 1.**
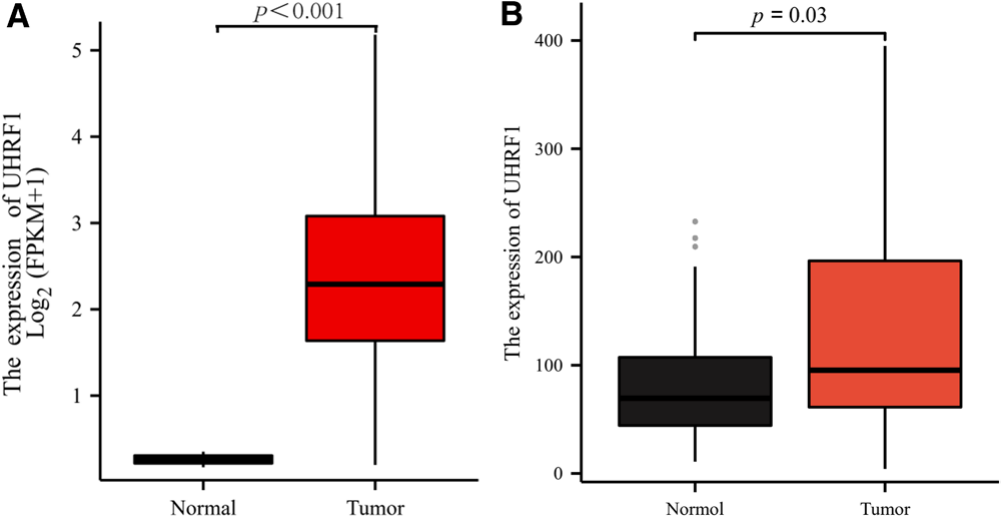
Comparison of UHRF1 expression in soft tissue sarcoma and paracancerous tissue (A) Differential expression of UHRF1 in TCGA database samples; (B) Differential expression of UHRF1 in GSE2719 samples. TCGA = the Cancer Genome Atlas.

Using the gene expression data of STS downloaded from TCGA, the Limma package tool in R software (4.0.4) was applied to analyze the expression level of UHRF1 in STS and adjacent tissues. As shown in Figure 1, the expression level of UHRF1 in STS was significantly higher than that in paracancerous tissues (*P* < .001). The GSE2719 dataset was downloaded from GEO database, and the expression level of UHRF1 in STS and adjacent tissues was analyzed. UHRF1 was differentially expressed in STS and adjacent tissues (*P* = .03).

### 3.3. Effect of UHRF1 expression level on prognosis of STS patients

**Figure 2.**
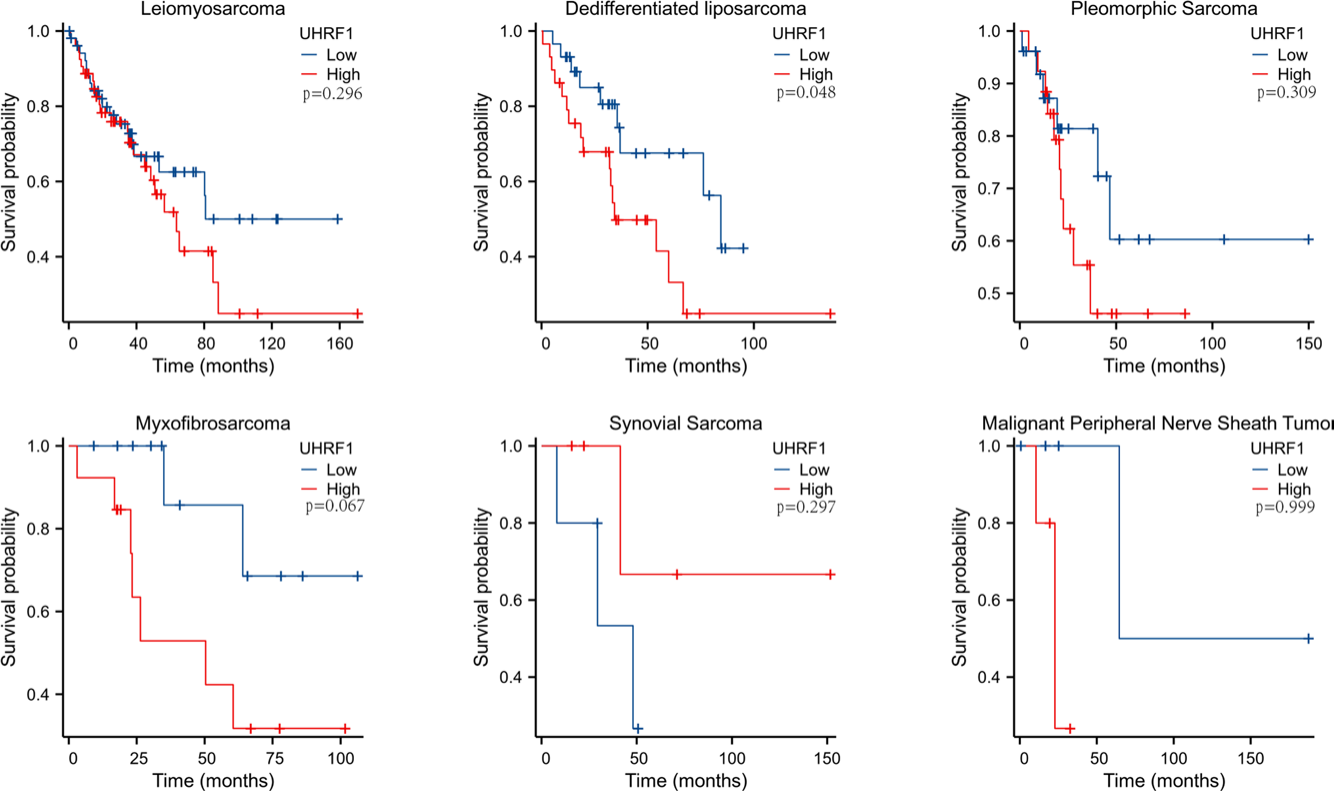
Survival status of patients with various subtypes of sarcoma with UHRF1 expression level analyzed by Kaplan–Meier method.

According to the median value of UHRF1 expression, STS patients were classified into high and low UHRF1 expression groups. The survival status of patients with STS with high UHRF1 expression and low UHRF1 expression was analyzed by Kaplan–Meier method (Fig. 2). As to leiomyosarcoma, dedifferentiated liposarcoma, pleomorphic sarcoma, myxfibrosarcoma, and MPNST, the OS in the UHRF1 high expression group was significantly shorter than that in the UHRF1 low expression group except for synovial sarcoma, and the difference was statistically significant in dedifferentiated liposarcoma (*P* = .046).

**Figure 3.**
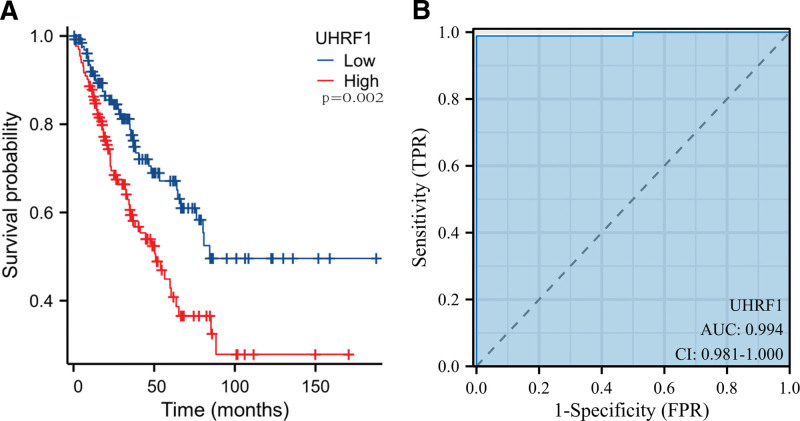
Relationship between UHRF1 expression level and prognosis in patients with soft tissue sarcoma (A) Kaplan–Meier curve; (B) Receiver Operating Characteristic curve.

Kaplan–Meier method analyzed the survival status of all STS patients with UHRF1 expression level, and the OS of patients with high UHRF1 expression level was significantly shorter than that of patients with low UHRF1 expression level (Fig. 3A, *P* = .002). Based on ROC analysis, the area ratio under ROC curve between STS tissue and adjacent tissue of UHRF1 expression was 0.994 (Fig. 3B). These data suggest that STS patients with high UHRF1 expression have a poor prognosis, which is a potential predictive molecular marker of STS prognosis.

The independent risk factors influencing the prognosis of STS patients were analyzed by Cox regression. Univariate Cox regression analysis revealed the number of tumors in STS patients (HR = 0.416, 95%CI = 0.260–0.666, *P* < .001), tumor depth (HR = 2.888, 95%CI = 0.910–9.168, *P* = .033), metastasis (HR = 2.888, 95% CI = 1.762–4.732, *P* < .001), residual tumor (HR = 2.637, 95%CI = 1.721–4.038, *P* < .001) and UHRF1 expression level (HR = 1.342, 95%CI = 1.105–1.630, *P* = .003) was significantly associated with OS. Multivariate Cox regression analysis indicated that UHRF1 was highly expressed (HR = 1.387, 95%CI = 1.008–1.907, *P* = .044), residual tumor (HR = 2.653, 95%CI = 1.385–5.081, *P* = .003), and metastasis (HR = 2.205, 95% CI = 1.188–4.092, *P* = .012) was an independent risk factor for prognosis in STS patients (Table 2).

**Table 2:** Cox regression analysis of independent risk factors affecting prognosis in patients with soft tissue sarcoma.

Characteristics	Univariate analysis		Multivariate analysis	
	Hazard ratio (95% CI)	*P* value	Hazard ratio (95% CI)	*P* value
Age	1.285 (0.864–1.911)	.214		
Gender	0.905 (0.607–1.349)	.622		
Tumor multifocal	0.416 (0.260–0.666)	<.001	0.649 (0.256–1.646)	.363
Tumor necrosis	1.367 (0.842–2.221)	.208		
Tumor depth	2.888 (0.910–9.168)	.033	3.770 (0.493–28.830)	.201
Histological type	1.081 (0.376–3.111)	.933		
Metastasis	2.888 (1.762–4.732)	<.001	2.205 (1.188–4.092)	.012
Residual tumor	2.637 (1.721–4.038)	<.001	2.653 (1.385–5.081)	.003
Radiation therapy	0.864 (0.557–1.339)	.508		
UHRF1	1.342 (1.105–1.630)	.003	1.387 (1.008–1.907)	.044

### 3.4. Nomogram predicts prognostic factors in STS patients

**Figure 4.**
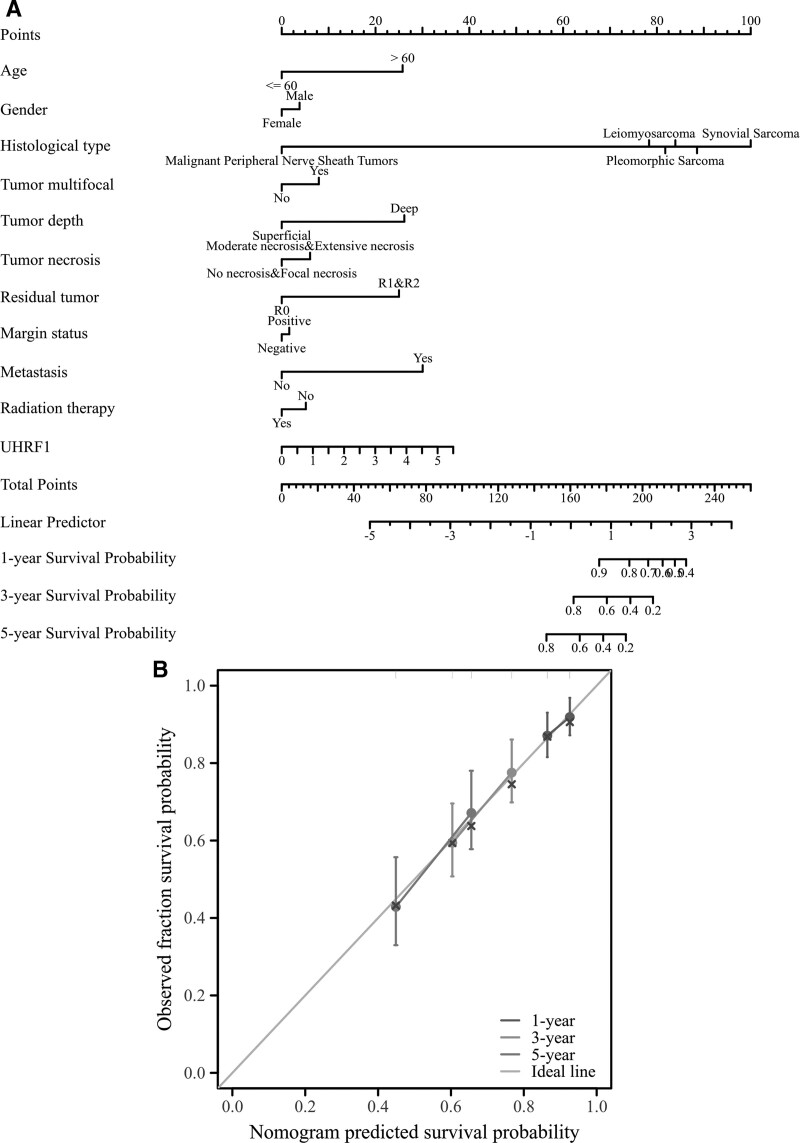
(A) Nomogram to predict the prognosis of patients with soft tissue sarcoma; (B) Calibration curve for predicting overall survival.

The relationship between clinicopathological factors and the prognosis of STS patients was comprehensively analyzed. The nomogram was drawn with R software, and different clinicopathological factors were assigned corresponding scores. These were summed to obtain the total score value, and a higher total score on the nomogram indicated a worse prognosis. The 1 -, 3 - and 5-year OS rates of STS patients were assessed on the basis of the total score. The nomogram (Fig. 4A) suggested that UHRF1 expression level and tumor histological type had a significant influence on the total score value, while other clinicopathological factors had relatively little influence on the total score. A bias correction line was constructed in the calibration plot to approximate the ideal curve (45°), which indicates perfect agreement between the predicted and observed values (Fig. 4B).

### 3.5. Functional enrichment analysis of UHRF1 gene

**Figure 5.**
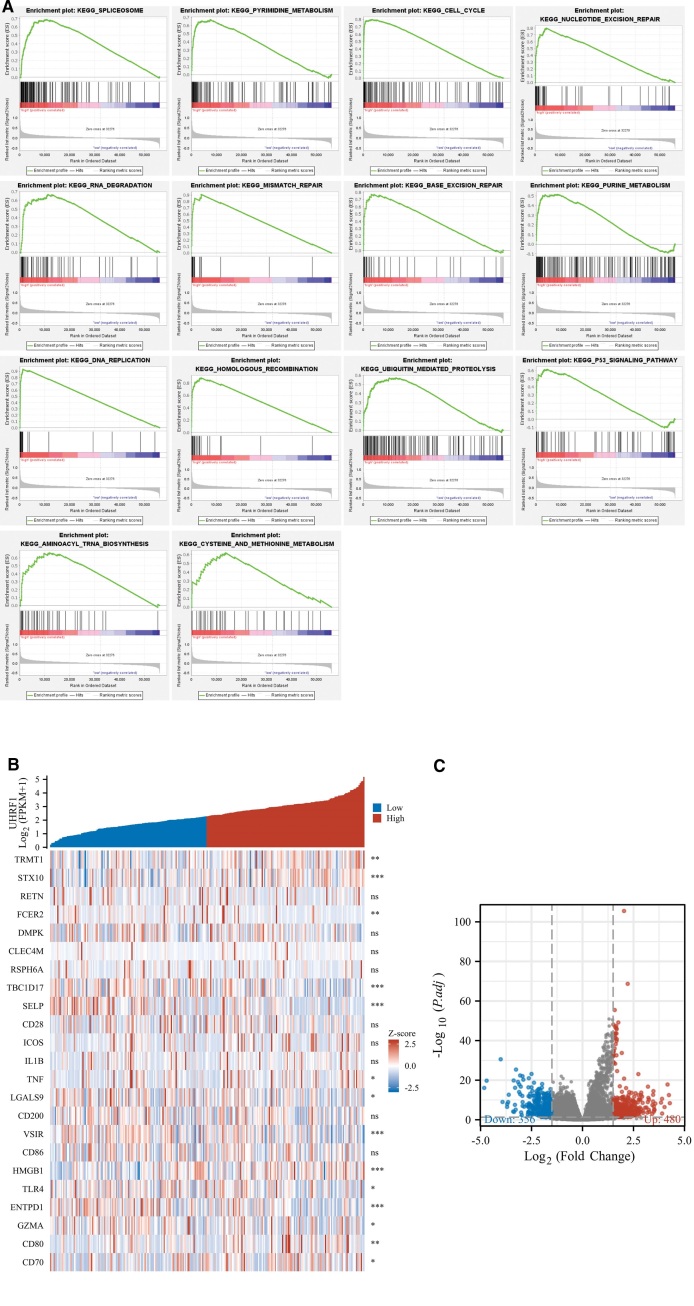
(A) Enrichment map of GSEA. (B) Heat map of co-expression of UHRF1 and immune-related genes in soft tissue sarcoma; (C) Volcano map for differential expression analysis of UHRF1 high-low expression group.

GSEA enrichment analysis suggested that UHRF1 was involved in 14 signaling pathways regulating mRNA spliceosome, pyrimidine metabolism, cell cycle, nucleotide excision repair, RNA degradation, DNA mismatch repair, base excision repair, purine metabolism, DNA replication, homologous recombination repair, protein ubiquitination, P53 signaling pathway, aminoacyl biosynthesis, cysteine and methionine metabolism were identified (Fig. 5A). The correlation between UHRF1 expression and immune-related genes in STS patients was explored by gene co-expression analysis. As is demonstrated in Figure 5B, UHRF1 expression was significantly correlated with the expression of immune checkpoint-related genes such as FCER2, SELP, TNF, LGALS9, VSIR, HMGB1, TLR4, ENTPD1, GZMA, CD80 and CD70. clusterPorfiler R package was applied to analyze the STS-related data in TCGA (adjusted *P* < .05 and log2FC ≥ 1.5), and the gene expression profiles of UHRF1 high expression group and low expression group were compared. A total of 836 DEG were identified in the UHRF1 high expression group, including 480 up-regulated genes and 356 down-regulated genes (Fig. 5C).

### 3.6. Analysis of PPI networks associated with UHRF1

**Figure 6.**
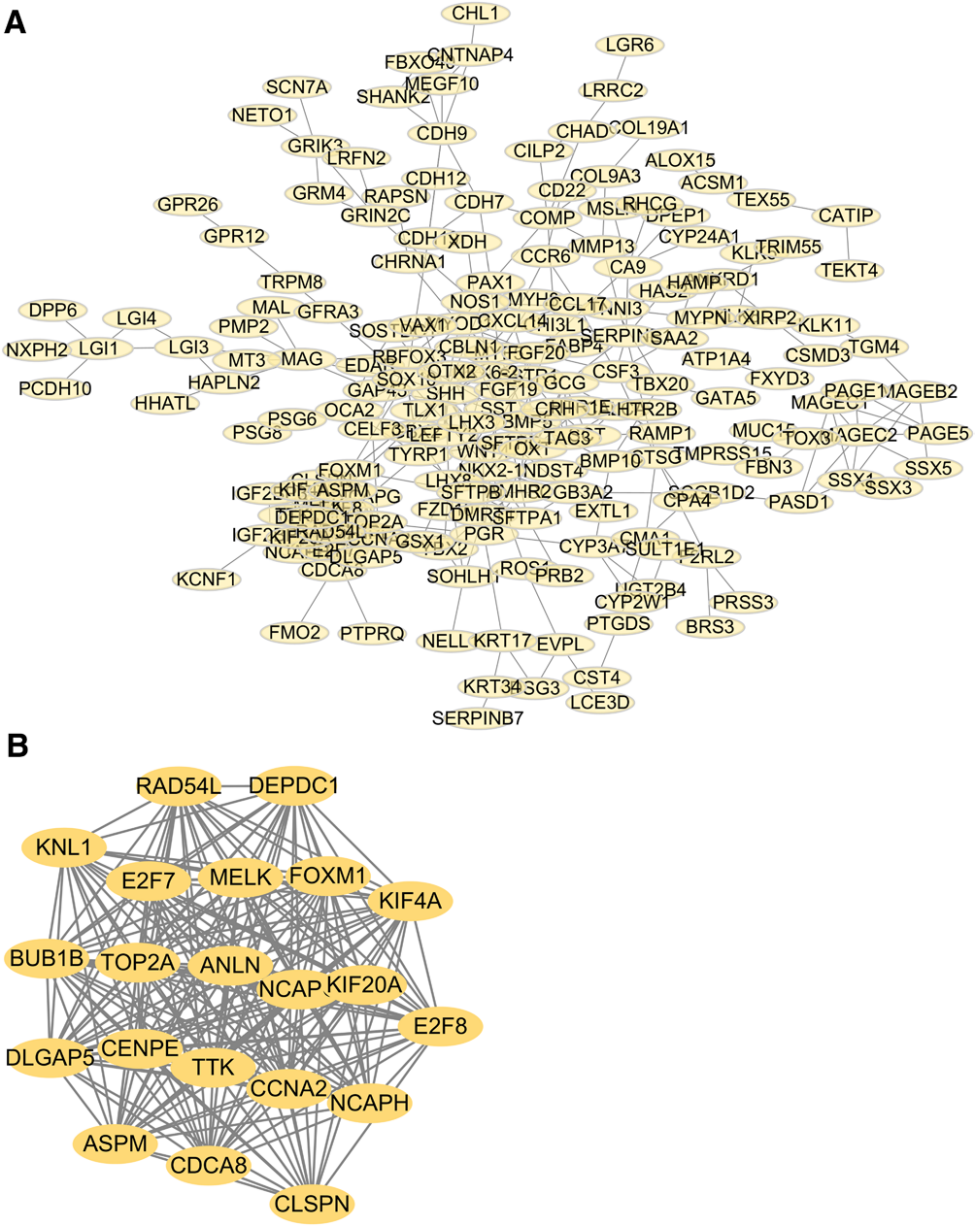
(A) Protein-protein interaction networks associated with UHRF1 gene in soft tissue sarcoma; (B) Hub gene network selected by MCODE toolkit by means of using Cytoscape software.

**Figure 7.**
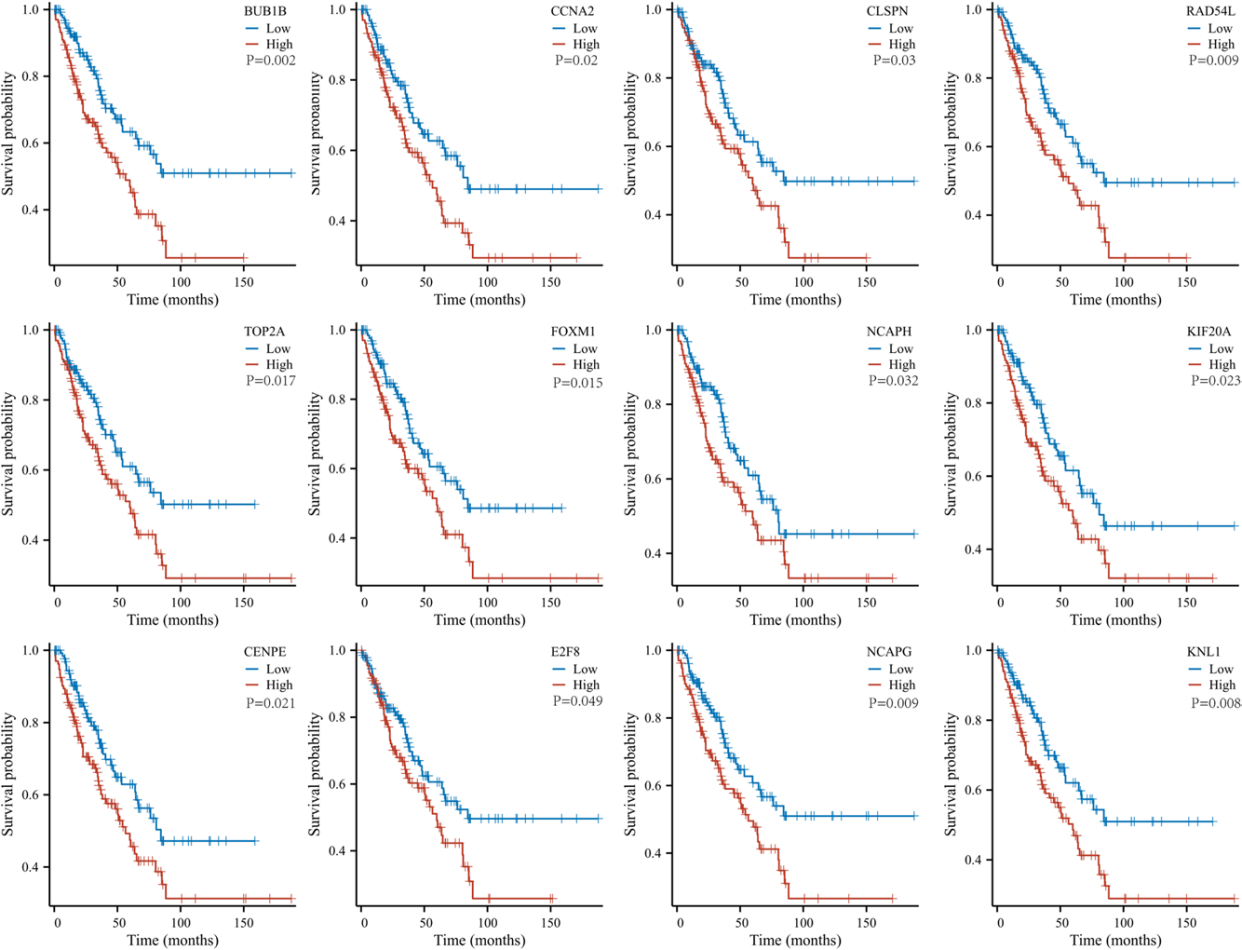
Relationship between the expression levels of 42 hub genes and Kaplan–Meier curve of overall survival in patients with soft tissue sarcoma.

For the sake of lucubrating the molecular basis of UHRF1 in STS, UHRF1 was imported into STRING database to obtain PPI network (Fig. 6A). After the input of UHRF1 gene, 193 nodes and 502 edges were obtained. The PPI network was further analyzed to screen for hub genes by means of using the MCODE tool in Cytoscape software. MCODE analysis demonstrated that the most significant module (MCODE score = 20.4) was composed of 21 hub genes (Fig. 6B). Among these hub genes, overexpression of BUB1B, CCNA2, CLSPN, RAD54L, TOP2A, FOXM1, NCAPH, KIF20A, CENPE, E2F8, NCAPG, KNL1 were significantly correlated with poor OS in STS patients (*P* < .05: Fig. 7).

### 3.7. Correlation between UHRF1 expression and immune cell infiltration

**Figure 8.**
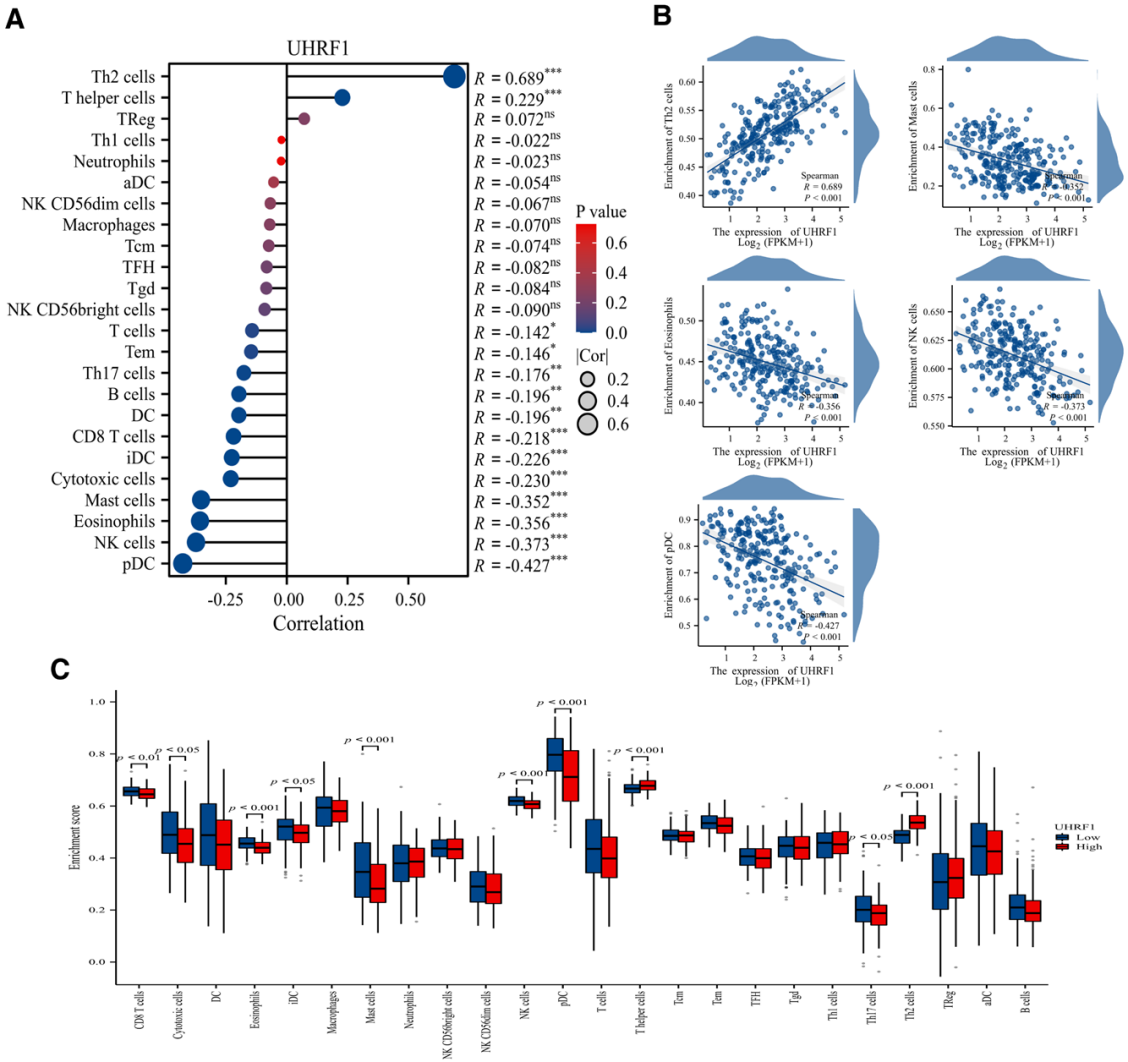
(A) Lollipop diagram of UHRF1 gene and 24 kinds of immune infiltrating cells; (B) Scatter plots of UHRF1 gene and 5 kinds of immune infiltrating cells with significant correlation; (C) Histogram of the relative infiltration level of 24 kinds of immune cells in UHRF1 gene high-low expression group.

The association between UHRF1 expression levels and immune cell infiltration levels quantified in STS was further explored by ssGSEA analysis. ssGSEA exhibited that the expression of UHRF1 in STS was positively correlated with the level of Th2 cell infiltration, and negatively correlated with plasmacytoid dendritic cells (pDC), natural killer cells (NK), Eosinophils, Mast cells (Fig. 8). Taken together, these findings reveal the biological processes and signaling pathways underlying the high expression of UHRF1, which are critical in the development and metastasis of tumors and may be potential targets for the treatment of STS.

## 4. Discussion

STS is a malignant tumor with many histological types occurring primarily in connective tissue and is a typical rare cancer, accounting for about 1% of all malignancies.^[3]^ STS is characterized by insidious onset, a low degree of differentiation and a high degree of malignancy. The 5-year OS and 5-year local recurrence rates for STS have been reported to be respectively 67.2-76% and 10–15%.^[20,21]^ At the time of diagnosis, 10% of STS patients have already developed distant metastases, lost the opportunity for radical surgery, and the 5-year survival rate drops to 15%.^[7]^ Hence, it is vital to explore novel biomarkers that can predict early diagnosis and prognosis in STS patients. The most widespread type of STS differentiation are liposarcoma, leiomyosarcoma, UPS, malignant peripheral nerve sheath tumor, and synovial sarcoma, which together occupy approximately 65% of all STS.^[5]^ Consistent with the results of this manuscript, the weight of STS was from large to small in order of histological type: leiomyosarcoma, dedifferentiated liposarcoma, pleomorphic sarcoma, myxofibrosarcoma, synovial sarcoma, and malignant peripheral nerve sheath tumor.

UHRF1 is a nucleoprotein gene associated with tumorigenesis. UHRF1 mainly exists in the S phase of the cell cycle and is closely relevant to cell proliferation. UHRF1 is overexpressed in various types of tumors, such as intrahepatic cholangiocarcinoma,^[22]^ bladder cancer,^[23]^ colorectal cancer,^[24]^ and lung adenocarcinoma.^[25]^ UHRF1 can regulate the expression of various oncogenes in malignancies. UHRF1 is overexpressed in esophageal squamous cell carcinoma tissues and is an independent prognostic factor for patients with esophageal squamous cell carcinoma.^[26]^ The research results of Wan et al^[27]^ indicated that UHRF1 was overexpressed in prostate cancer cells. Furthermore, by knocking down UHRF1 expression with specific siRNA contribute to UHRF1 expression was down-regulated, inhibiting the proliferation and migration of prostate cancer cells and ultimately inducing apoptosis. Kim et al^[28]^ found through immunohistochemical detection that compared with adjacent tissues of cervical cancer, UHRF1 was overexpressed in cervical cancer, and the mechanism was that UHRF1 mediated TXNIP promoter methylation, reduced the expression of tumor suppressor genes, and promoted cancer-cell-growth.

In this manuscript, the analysis using the TCGA database revealed that the expression of UHRF1 in STS was significantly higher than that in adjacent tissues. The analysis of the GSE2719 dataset downloaded from the GEO database once again verified this result. Correlation analysis of clinicopathological factors found that UHRF1 expression was significantly associated with tumor necrosis, histological type, and distant metastasis in STS patients. In this manuscript, the survival prognosis of patients with high and low UHRF1 expression was analyzed, and the results indicated that the OS time of patients with high UHRF1 expression was shorter than that of patients with low UHRF1 expression. Besides, this study used TCGA data to construct a nomogram for predicting the prognosis of STS patients, which more intuitively understood the importance of UHRF1 expression level in predicting the prognosis of STS patients. Cox regression univariate analysis suggested that the number of tumors, tumor depth, metastasis, and residual tumors in STS patients were significantly correlated with OS. Cox regression multivariate analysis revealed that high UHRF1 expression was an independent risk factor affecting the prognosis of STS patients. In conclusion, the manuscript results manifest that high UHRF1 expression indicates poor prognosis in STS patients, and it may become a potential molecular marker for predicting the prognosis of STS patients.

For the purpose of exploring the potential molecular mechanism of UHRF1 in the progression of STS, this study used standardized RNA-Seq data obtained from TCGA for GSEA enrichment analysis. The results suggest that the STS samples of the UHRF1 gene high expression group are mainly enriched in 14 key pathways regulating mRNA spliceosome, pyrimidine metabolism, cell cycle, nucleotide excision repair, RNA degradation, DNA mismatch repair, base excision repair, purine metabolism, DNA replication, homologous recombination repair, protein ubiquitination, P53 signaling pathway, aminoacyl biosynthesis, cysteine and methionine metabolism.

Overexpression of UHRF1 can accelerate the proliferation or metabolism of cancer cells, and downregulation of UHRF1 can induce cell cycle arrest, DNA damage response and apoptosis. Tu et al^[25]^ observed that the downregulation of UHRF1 gene in lung adenocarcinoma (ADC) cells can increase G1 phase, decrease G2 phase, and induce apoptosis. Therefore, the upregulation of UHRF1 in lung ADC is accompanied by poor OS rate. UHRF1 is a pivotal regulatory protein of DNA methylation and histone.^[29]^ UHRF1 recruits the DNA methyltransferase DNMT1 to newly synthesized DNA, which plays a vital role in maintaining DNA methylation, and methylation modification is the key to transmitting epigenetic information during cell division.^[30]^ In esophageal squamous cell carcinoma cells, knockdown of UHRF1 expression can lead to overall DNA hypomethylation, inhibit the proliferation of cancer cells, induce cell cycle arrest in G2/M phase, accompanied by the activation of DNA damage response pathway, and ultimately induce apoptosis.^[27]^ Zhu et al^[22]^ discovered that UHRF1 was overexpressed in intrahepatic cholangiocarcinoma (ICC) tissues, and down-regulated UHRF1 weakened the G1/S cell cycle transition, thereby inhibiting cell proliferation and tumor growth.UHRF1 can act as an E3 ubiquitin ligase and has ubiquitination function. The ubiquitination modification of histones can also affect the methylation process of DNA. Nishiyama et al^[31]^ discovered that H3K23 ubiquitination dependent on UHRF1 coupled DNA methylation with DNA replication. The cyclic domain of UHRF1 has a single ubiquitination function, and modifying H3K23 in the S phase of the cell cycle helps DNMT1 targeted recruitment to UHRF1.^[31]^ By mediating the activation of the UHRF1-DNMT1 axis, it promotes the proliferation of cancer cells.^[32]^ Zhou et al^[33]^ found that UHRF1 was underexpressed in osteogenic differentiation, but overexpressed in osteosarcoma. Furthermore, further studies hinted that UHRF1 existed in nuclear chromatin, combined with transcription-related proteins, and mediated protein degradation through ubiquitination, regulating DNA repair and cell cycle, and promoting the proliferation of osteosarcoma. UHRF1 was overexpressed in renal cell carcinoma tissues, and further exploration discovered that UHRF1 can promote non-degrading ubiquitination of p53, inhibit the p53 pathway, and assist renal cell carcinoma cells to get rid of p53-dependent apoptosis.^[34]^ Li et al^[35]^ also reported that UHRF1 participates in the regulation of the p53 pathway in HCC cells, and is correlated with the unfavorable prognosis of HCC. This manuscript obtained consistent results with the above studies through GSEA enrichment analysis. GSEA enrichment analysis showed that high expression of UHRF1 may promote the progression of STS by regulating cell cycle, nucleotide excision repair, DNA mismatch repair, base excision repair, protein ubiquitination, P53 signaling pathway and other crucial pathways, resulting in unfavorable prognosis of STS patients.

Cancer immunotherapy is an extraordinarily successful and rapidly developing treatment method, and immunotherapy has gradually become an important treatment option for patients with various types of cancer. It has been reported that the level of immune cell infiltration affects the efficacy of immunotherapy.^[36]^ There have been reports that the utilization of immune checkpoint inhibitors and anti-angiogenic immunotherapy can enhance the prognosis of STS patients.^[37]^ However, the research on predicting and prognostic biomarkers for STS patients is still limited, and further research is requisite. Currently, there is no evidence to revealing whether the expression of UHRF1 is connected with immune cell infiltration in STS. Previous studies have found that UHRF1 is abundant in immune cells, such as neutrophils, eosinophils, dendritic cells, macrophages, NK.^[35,38,39]^ The ssGESA analysis in this manuscript also indicated that UHRF1 expression was associated with Th2 cells, TH17 cells, helper T cells, pDC, NK, mast cells, immature dendritic cells, eosinophils, cytotoxic cells, CD8 + T cells and other immune cells. UHRF1 overexpression was significantly associated with the expression of immune checkpoint-related genes FCER2, SELP, TNF, LGALS9, VSIR, HMGB1, TLR4, ENTPD1, GZMA, CD80, and CD70. In addition, a UHRF1-related PPI network was constructed, and 21 hub genes were ultimately screened. BUB1B, CCNA2, CLSPN, RAD54L, TOP2A, FOXM1, NCAPH, KIF20A, CENPE, E2F8, NCAPG, and KNL1 genes had a significant impact on the OS of HCC patients.

There are certain limitations in this manuscript. First, this manuscript is based on public databases, and clinical data need to be expanded to verify the effect of UHRF1 on the prognosis of STS patients. Secondly, further studies are essential to elucidate the biological function of UHRF1 expressed in cell lines and animal models.

In conclusion, this manuscript utilized TCGA data for bioinformatics analysis and discovered that UHRF1 was overexpressed in STS, indicating poor prognosis of STS patients. The expression level of UHRF1 was an independent risk factor affecting the prognosis of STS patients, suggesting that UHRF1 may become a potential molecular marker for predicting the prognosis of STS patients. UHRF1 may be involved in regulating key pathways such as cell cycle, nucleotide excision repair, RNA degradation, DNA mismatch repair, base excision repair, protein ubiquitination, and P53 signaling pathway. Functional enrichment analysis indicated that the immune regulation of UHRF1 was involved in the occurrence and development of STS. Our results hint that UHRF1 may provide an innovative thread for the pathogenesis, molecular targeted therapy, and prognosis prediction of STS.

## Author contributions

**Conceptualization:** Qiang Shu, Xu Bo.

**Data curation:** Qiang Shu, XiaoLing Liu, Xing Xiang.

**Formal analysis:** Qiang Shu, XiaoLing Liu.

**Methodology:** Qiang Shu, XiaoLing Liu, Xing Xiang.

**Project administration:** Xu Bo.

**Software:** Qiang Shu.

**Supervision:** Xing Xiang, Xu Bo.

**Validation:** Xing Xiang, Xu Bo.

**Visualization:** Xu Bo.

**Writing – original draft:** Qiang Shu.

**Writing – review & editing:** Qiang Shu, Xing Xiang.
